# Persistence of Coxsackievirus B4 in Pancreatic β Cells Disturbs Insulin Maturation, Pattern of Cellular Proteins, and DNA Methylation

**DOI:** 10.3390/microorganisms9061125

**Published:** 2021-05-22

**Authors:** Magloire Pandoua Nekoua, Antoine Bertin, Famara Sane, Jean-Pascal Gimeno, Isabelle Fournier, Michel Salzet, Ilka Engelmann, Enagnon Kazali Alidjinou, Didier Hober

**Affiliations:** 1 Laboratoire de Virologie ULR3610, Université de Lille, CHU Lille, F-59000 Lille, France; magloire-pandoua.nekoua@univ-lille.fr (M.P.N.); antoinebertin@laposte.net (A.B.); famara.sane@chru-lille.fr (F.S.); ilka.engelmann@chru-lille.fr (I.E.); enagnonkazali.alidjinou@chru-lille.fr (E.K.A.); 2 Laboratoire Protéomique, Réponse Inflammatoire et Spectrométrie de Masse (PRISM), Inserm U1192, Université de Lille, F-59000 Lille, France; jean-pascal.gimeno@univ-lille.fr (J.-P.G.); isabelle.fournier@univ-lille.fr (I.F.); michel.salzet@univ-lille.fr (M.S.)

**Keywords:** coxsackievirus B4, persistence, insulin, pro-hormone convertase 2, DNA methylation, pancreatic β cell, in vitro, INS-1 cell line, type 1 diabetes

## Abstract

Coxsackievirus-B4 (CV-B4) can persist in pancreatic cell lines and impair the phenoytpe and/or gene expressions in these cells; however, the models used to study this phenomenon did not produce insulin. Therefore, we investigated CV-B4 persistence and its consequences in insulin-producing pancreatic β cells. The insulin-secreting rat β cell line, INS-1, was infected with CV-B4. After lysis of a large part of the cell layer, the culture was still maintained and no additional cytopathic effect was observed. The amount of insulin in supernatants of cell cultures persistently infected with CV-B4 was not affected by the infection; in fact, a larger quantity of proinsulin was found. The mRNA expression of pro-hormone convertase 2, an enzyme involved in the maturation of proinsulin into insulin and studied using real-time reverse transcription-polymerase chain reaction, was inhibited in infected cultures. Further, the pattern of 47 cell proteins analyzed using Shotgun mass spectrometry was significantly modified. The DNA of persistently infected cell cultures was hypermethylated unlike that of controls. The persistent infection of INS-1 cells with CV-B4 had a deep impact on these cells, especially on insulin metabolism. Cellular changes caused by persistent CV-B4 infection of β cells can play a role in type 1 diabetes pathogenesis.

## 1. Introduction

Type 1 diabetes (T1D) is a metabolic disease characterized by chronic hyperglycemia, resulting from defective production of insulin due to the selective destruction or loss of functional insulin-producing β cells through an autoimmune process occurring in genetically predisposed individuals. Insulin synthesis and secretion by pancreatic β cells are highly regulated by intracellular mechanisms, which are critical for maintaining the quality and quantity of this anabolic hormone and blood glucose homeostasis [1]. The maturation of insulin in β cells requires the cleavage of proinsulin by pro-hormone convertase 1 and 2 (PC1 and 2) encoded by the proprotein convertase subtilisin/kexin type 1 (PCSK1) and 2 (PCSK2) gene, respectively, resulting in the formation of mature insulin, and C-peptide [2,3]. The altered biological activity of insulin in diabetes is reported to be a consequence of proinsulin misfolding; insulin gene mutation; impaired insulin gene transcription; and perturbed proinsulin-to-insulin conversion by inflammatory cytokines such as IL-1β, interferon-γ (IFN-γ), and IFN-α [4,5].

Enteroviruses, especially coxsackieviruses-B (CV-B), are the most suspected environmental factors associated with the development of islet autoimmunity or the onset and progression of T1D [6]; however, the underlying mechanisms leading to defective synthesis or secretion of insulin in pancreatic β cells remain an open issue. Coxsackieviruses-B (CV-B1–6) are small, non-enveloped, positive-sense single-stranded RNA genome viruses belonging to the *Enterovirus* genus of the *Picornaviridae* family [7]. These ubiquitous pathogens are transmitted mainly through the fecal–oral and respiratory routes and are involved in several mild or severe acute clinical infections or in chronic diseases such as chronic myocarditis, dilated cardiomyopathy, and T1D [6,8,9,10]. The frequent detection of enteroviral RNA and capsid protein VP1 in the blood, intestine, pancreatic islets, and especially β cells of diabetic patients supports the hypothesis of the role of persistent infection as a major mechanism in the pathogenesis of CVB-related T1D [11,12,13,14,15,16]. CV-B can induce cytolytic infection in human pancreatic islet β cells [17,18,19]; additionally, they can establish a persistent infection in vitro as well as in vivo [20]. The persistence of CV-B is thought to result from a coevolution over a prolonged period between the host cell and the virus, leading to changes in some of their characteristics [21]. During CV-B persistence, viral genomic modifications such as mutations with amino acid substitutions [22,23], formation of a stable double-stranded RNA genomic form [24], and a deletion in the 5′ non-coding region [25,26] have been reported. A study using quantitative mass spectrometry-based proteomics revealed that HIV-1 infection can activate a molecular inhibitor of cell apoptosis, BIRC5, leading to long-term survival of infected CD4+ cells and persistence of the virus in infected individuals [27].

A persistent CV-B4 infection was established in human pancreatic β cells in vitro. It was characterized by a low proportion of infected cells, low levels of viral load, and production of significant levels of IFN-α [28,29], which can promote inflammation and bystander activation of anti-β cell autoreactive T lymphocytes, leading to autoimmune diabetes [30,31]. Several structural or functional alterations of pancreatic cells have also been reported during persistent infection with CV-B4, which can play a role in the development of T1D. We previously demonstrated that the persistence of CV-B4 E2 in human pancreatic ductal-like cells leads to a decrease in the expression of viral receptors [23], a disruption of microRNA profiles [23,32], an alteration of expression of the transcription factor Pdx-1 required for the formation of the endocrine pancreas, and an impaired differentiation of these cells into islet-like cell aggregates (ICA) [23,33]. Thus, we demonstrated that CV-B4 was able to persist in pancreatic cell lines and impair the phenoytpe and/or the expression of genes in these cells; however, these models did not produce insulin. Therefore, in this study, we aimed to investigate the persistence of CV-B4 in insulin-producing pancreatic β cell line, INS-1, and its consequences.

## 2. Materials and Methods

### 2.1. Cell Lines and Virus

The β cell line, INS-1, is derived from a rat insulinoma induced by x-ray irradiation of New England Deaconess Hospital (NEDH) inbred albino rats [34,35] and was kindly provided by Professor A. Abderrahmani (UMR 8199, Université de Lille). The INS-1 cell line was cultured in Roswell Park Memorial Institute (RPMI)-1640 medium (Gibco^®^, Invitrogen, Paisley, UK) supplemented with 10% inactivated fetal calf serum (FCS), 100 U/mL penicillin and 100 μg/mL streptomycin, 1% L-glutamine, 1% sodium pyruvate (Invitrogen, Saint Aubin, France), and 50 μM 2-mercaptoethanol (Sigma-Aldrich, Saint-Quentin Fallavier, France). The HEp-2 cell line (BioWhittaker, Verviers, Belgium) was cultured in Eagle’s Minimum Essential Medium supplemented with 10% FCS, 100 U/mL penicillin and 100 μg/mL streptomycin, and 1% L-glutamine. The diabetogenic strain CV-B4 E2 was provided by Ji-Won Yoon (Julia McFarlane Diabetes Research Center, Calgary, AB, Canada) and was propagated in HEp-2 cells as previously described [33,36]. The viral titers in supernatants were assessed on HEp-2 cell monolayers using the end-point dilution assay. The cytopathic effect (CPE) was then read using a CKX41 phase contrast microscope (Olympus, Rungis, Fance) and the Spearman–Karber method was used to determine the 50% tissue culture infectious dose (TCID_50_). The results are expressed as TCID_50_/mL. Virus stocks were stored at −80 °C.

### 2.2. Chronic Cell Infections and Viral Progeny in Supernatants

INS-1 cells were harvested and seeded at 1.25 × 10^5^ cells per well in 24-well plates and were inoculated with CV-B4 E2 at a multiplicity of infection (MOI) of 0.01. Twenty-four hours post-infection (p.i) at 37 °C and 5% CO_2_, cells were rinsed three times with RPMI-1640 medium and every 3 days during the acute lytic infection. CV-B4 E2-infected and mock-infected cell cultures were then seeded and sub-cultured in cell culture flasks with vented caps (Becton–Dickinson, Le Pont de Claix, France), and culture supernatants were harvested throughout the monitoring of infection and stored at −80 °C.

### 2.3. Cell Viability Assay

The cellular metabolic activity of CV-B4 E2-infected and mock-infected INS-1 cells was assessed using Uptiblue™ viable cell counting reagent (Uptima, Interchim^®^, Montluçon, France) according to the manufacturer’s instructions. Briefly, the cells were incubated for 6 h at 37 °C, 5% CO_2_ with Uptiblue. The supernatant was collected, and the absorbance was read at 570 nm and 600 nm using a Multiskan GO microplate reader (Thermo Fisher Scientific, Illkirch, France). The difference between the absorbances at the two wavelengths constitutes the viability index of the culture. The viability of these cells were also evaluated under a microscope using the Trypan blue dye exclusion test.

### 2.4. DNA and RNA Extraction

Cells were scraped and collected in 350 µL of RLT Plus Lysis buffer (Qiagen^®^, Courtaboeuf, France) before simultaneous purification of genomic DNA and total RNA using the AllPrep DNA/RNA Mini Kit (Qiagen^®^) according to the manufacturer’s instructions. The purified DNA and RNA were quantified by reading absorbance at 260 nm and 280 nm with a µDrop^®^ plate and spectrophotometer (Thermo Fisher Scientific).

### 2.5. One-Step RT-PCR and Agarose Gel Electrophoresis

The intracellular positive enteroviral RNA strand from CV-B4 E2-infected INS-1 cells was detected using a specific one-step real-time polymerase chain reaction (RT-PCR). The β-actin gene was co-amplified as a control using the SuperScript™ One-step RT-PCR (Thermo Fisher Scientific). Primers used for the EV genome and β-actin gene are shown in Table 1.

The reactions were performed using an Applied GeneAmp PCR System 2400 thermocycler (Perkin Elmer, Villebon sur Yvette, France) according the following program: 30 min at 50 °C for reverse transcription and 2 min at 94 °C for initial denaturation; then, 35 PCR cycles consisting of 94 °C for 30 s, 55 °C for 45 s, and 68 °C for 45 s. A terminal elongation of 10 min at 68 °C was then carried out, before storing the samples at 4 °C. The specific amplicons (435 bp for CV-B4 and 101 bp for β-actin) were analyzed on a 2% agarose gel containing 0.5 μg/mL ethidium bromide (Sigma-Aldrich) and visualized using the Gel Doc 2000 system (Bio-Rad, Marnes-la-Coquette, France).

### 2.6. Real-Time Quantitative PCR

PCSK2 mRNA was quantified using RT-PCR on Mx3000p^®^ (Stratagene, Santa Clara, CA, USA) with the SYBR^®^ Green SuperMix (Bio-Rad Laboratories), according to the manufacturer’s instructions. The SuperScript^®^ Double-Stranded cDNA synthesis kit (Invitrogen^TM^) was used to perform the reverse transcription, using oligo-dT as the reverse transcription primer, according to the manufacturer’s instructions. The primer sequences used for PCSK2 mRNA are listed in Table 1. The β-actin gene was used as endogenous control for normalization. PCSK2 mRNA relative expression in mock versus CV-B4 E2-infected INS-1 cells was determined with the 2^−ΔΔCt^ formula [37]. All reactions were performed in triplicates.

### 2.7. Quantification of Insulin, Proinsulin, and C-Peptide

The supernatant of INS-1 cell cultures was recovered at various times post-inoculation. Insulin and proinsulin were quantified using Mercodia^®^ mouse ELISA kits and C-peptide was quantified using a mouse C-Peptide ELISA Kit (Aviva Systems Biology, San Diego, CA, USA), according to the manufacturer’s instructions.

### 2.8. Immunofluorescence Assay

Mock-infected and CV-B4-infected INS-1 cells were seeded on sterile glass slides in 24-well plates. The cells were then fixed with 4% paraformaldehyde and permeabilized with 0.1% Triton X-100 (Sigma-Aldrich). The presence of intracellular enteroviral capsid protein VP1 and insulin in INS-1 cells was investigated using an immunofluorescence assay as previously described [29,33]. Briefly, permeabilized cells were incubated overnight with a mouse anti-VP1 antibody, clone 5D8/1 (DAKO, Les Ulis, France) and a guinea pig anti-insulin antibody (DAKO, Via Real, CA, USA). The respective secondary antibodies: Alexa Fluor™ 488-conjugated rabbit anti-mouse antibody and Alexa Fluor™ 594-conjugated goat anti-guinea pig (Abcam, Cambridge, UK) were then applied sequentially for 1 h at room temperature. Cell nuclei were then stained with Hoescht dye solution, (Invitrogen^TM^) for 15 min. The slides were observed under a Leica DMi8 fluorescence microscope (Leica Microsystemes, Nanterre, France) at ×40, and positive cells were visualized with specific detector filters.

### 2.9. Quantification of DNA Methylation

Global DNA methylation was quantified by specifically measuring levels of 5-methylcytosine (5-mC) with the MethylFlash Methylated DNA 5-mC Quantification Kit (Epigentek, Farmingdale, NY, USA). Briefly, 100 ng DNA from each sample were bound to a 96-well ELISA-like microplate in duplicate and the methylated fraction of DNA was detected using capture and detection antibodies at 1:1000 and 1:2000 dilution, respectively, and at 1:5000 dilution of enhancer solution. The signal detection was performed by adding a developer solution, and optical density (OD) was read at 450 nm with a microplate reader Multiskan GO (Thermo Fisher Scientific). The amount of methylated DNA was proportional to the OD intensity measured. The percent methylation was calculated according to the following Equation (1):

5-mC% = ([{Sample OD − ME3 OD}/S]/[{ME4 OD − ME3 OD} × 2/P]) × 100(1)

where ME3 = unmethylated DNA (negative control), S = amount of input sample DNA in nanograms, ME4 = methylated DNA (positive control) and P = amount of input positive control (ME4) in nanograms

### 2.10. Protein Extraction and Digestion

Samples were directly subjected to in-solution tryptic digestion. The sample was first incubated with a denaturation buffer (urea, 6 M), and sonication (3 × 1 min) was performed. The sample was reduced by the addition of 1,4 dithiothreitol (40 mM) and incubated at 55 °C for 40 min. Then, the sample proteins were alkylated by adding iodoacetamide at a final concentration of 275 mM (V = 50 µL) and incubating for 40 min in the dark. Thiourea (600 mM) was added, and trypsin (40 µg/mL) was finally added for a last incubation overnight at 37 °C. The digestion was stopped with formic acid at 2% (*v*/*v*). Sample was subjected to desalting using the C18 ZipTip pipette tip (Millipore, Darmstadt, Germany), and concentrated peptides were dried under vacuum (Speedvac SPD131DDA, Thermo Scientific). All reagents were purchased from Sigma-Aldrich (Saint-Quentin Fallavier, France).

### 2.11. Mass Spectrometry Data Acquisition UPLC-MS/MS

The digested peptides were reconstituted with an acetonitrile/formic acid solution (2%/0.1%) (Biosolve B. V. Valkensvaard, Netherlands/Fluka^TM^). Samples were separated at a flow rate of 300 nL/min, by an online reversed-phase chromatographic system (Easy-nLC 1000 UPLC system, Thermo Scientific, Waltham, MA, USA) equipped with a Proxeon trap column (100 µm internal diameter × 2 cm, Thermo Scientific) and a C_18_ packed capillary column (Acclaim PepMap 100 C18, 75 µm ID × 50 cm, Thermo Scientific). The LC eluent was electrosprayed directly from the analytical column, and a voltage of 1.7 kV was applied via the liquid junction of the nanospray source. The chromatographic system was interfaced to a Q Exactive mass spectrometer (Thermo Scientific) set to acquire a top 10 MS^2^ in a data-dependent mode. The survey scans were conducted at a resolving power of 70 000 FWHM (*m*/*z* 400) in the positive mode and using an automatic gain control target of 5 × 10^6^. Default charge state was set at 2, unassigned and +1 charge states were rejected, and dynamic exclusion was enabled for 20 s. The scan range was set to 300–1600 *m*/*z*. For ddMS^2^, the scan range was 200–2000 *m*/*z*; one microscan was acquired at 17,500 FWHM, and an isolation window of 4.0 *m*/*z* was used.

### 2.12. Data and Statistical Analyses

Raw files were processed during SEQUEST version1.4.114 (Proteome Discoverer, Thermo Fisher Scientific). The protein identifications were obtained using the following parameters for interrogation: Parent Mass tolerance: ±10 ppm, Fragment Mass tolerance: 0.6 Da, Max. ΔCn = 0.05, Dynamic Modification: Oxidation/+15.995 Da (methionine), Enzyme: trypsin, 2 miss cleavages, Protein FDR = 0.01. The searches were Static Modification: Carbamidomethylation/+57 Da (cysteine) performed using the UniprotKB/Swiss-Prot database (accessed September 16, 2014) filtered with *Rattus norvegicus* (41604 sequences), and coxsackievirus B4 (Q8687 uniprot 2017) taxonomy using the SEQUEST HT algorithm. In general, peptide identifications were accepted if they could be established at a given probability to achieve an FDR less than 1.0%, while protein identifications were accepted if they could be established at a given probability to achieve an FDR less than 1.0% and contained at least two identified peptides. Data analyses were performed using Graph Pad Prism 6.0 (Graph Pad Inc., San Diego, CA, USA). Results are presented as mean ± standard deviation. Comparisons were performed using Student’s t or Mann Whitney U tests when appropriate. *p* values < 0.05 were considered to indicate statistically significant differences.

## 3. Results

### 3.1. Persistent CV-B4 E2 Infection of Pancreatic β Cells

**Figure 1:**
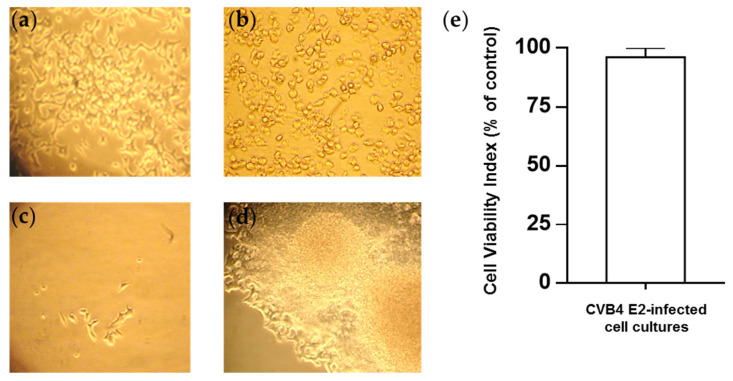
Infection of pancreatic β cells by CV-B4 E2. Rat pancreatic β cells (INS-1 cell line) (**a**) infected with CV-B4 E2 at an MOI of 0.01 (**b**) The cytopathic effect (CPE) observed 72 h p.i, followed by lysis of most cells. (**c**) Only a few cells survived the lytic phase of infection. (**d**) Culture was maintained, and the cells organized into clusters. The cells were observed under an inverted light microscope (magnification ×100). (**e**) The viability of the cells was evaluated using the Uptiblue™ assay. The results are expressed as % relative to mock-infected cells (for which the viability index is 100%). The values correspond to the means ± SD of 10 independent experiments.

The β cell line, INS-1, were infected with CV-B4 E2 at an MOI of 0.01 (Figure 1a). After 72 h of incubation, massive cell lysis was observed (Figure 1b). However, the culture was still maintained. Cell growth was slowed down, and less than 1% of the surface of the culture flask was covered with cells on day 15 p.i. (Figure 1c). Then, cell growth was faster, and cells organized themselves into clusters (Figure 1d). The cell viability values of CV-B4 and mock-infected INS-1 cells monitored through metabolic activity, evaluated using Uptiblue™ assay, were not significantly different from those after the acute lytic infection (Figure 1e).

**Figure 2:**
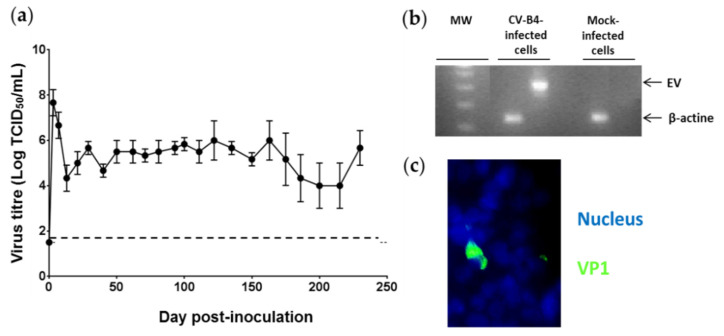
Persistence of CV-B4 E2 in cultures of pancreatic β cells. Cultures of pancreatic β cells (INS-1 cell line) infected with CV-B4 E2 were monitored over time. Culture supernatant and cells were recovered. (**a**) Infectious viral particles detected via titration in the culture supernatant. The limit of detection of the infectious titer (1.5) is indicated by the dotted line. The values are the mean ± SD of three independent experiments. (**b**) Intracellular enteroviral RNA detected using RT-PCR, followed by agarose gel electrophoretic migration of the amplification products. MW: Size marker (DNA Ladder 100 bp ^®^). A 435-bp (EV) band corresponds to the amplification product of enteroviral (EV) RNA obtained through RT-PCR. β-actin was used as a control for the presence of RNA and corresponds to a band of 182 bp. (**c**) CV-B4-infected INS-1 cells were stained for intracellular VP1 with mouse anti-enterovirus VP1 and Alexa Fluor™ 488-conjugated rabbit anti-mouse antibody using an indirect immunofluorescence assay. The nuclei were stained with Hoechst Dye 33342^®^ (original magnification ×20). Pictures from one representative experiment out of three are shown.

Titers of infectious particles were up to 8 logs TCID_50_/mL in the acute phase, then ranging from 3.5 to 6 logs TCID_50_/mL were found in supernatants of CV-B4 E2-infected cells throughout the culture period until day 230 p.i. (Figure 2a). The presence of intracellular enteroviral RNA in infected cells was detected using RT-PCR (Figure 2b). The viral capsid protein, VP1, of CV-B4 was detected in 1% of infected INS-1 cells on day 45 p.i. using an immunofluorescence assay (Figure 2c).

### 3.2. Persistent CV-B4 E2 Infection of Pancreatic β Cells Results in Change in Insulin Metabolism

**Figure 3:**
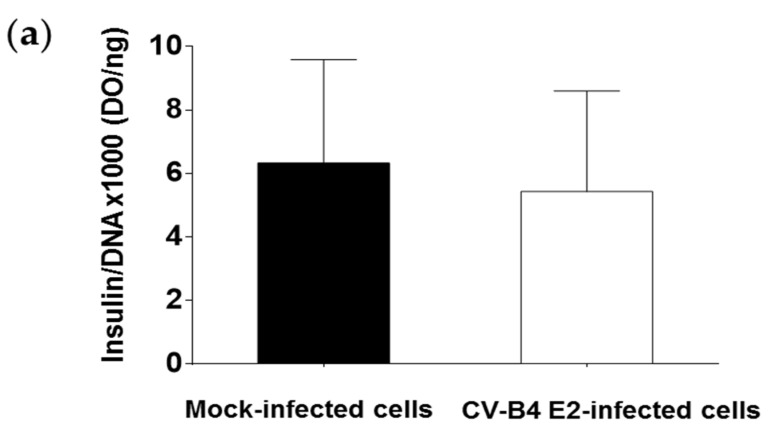

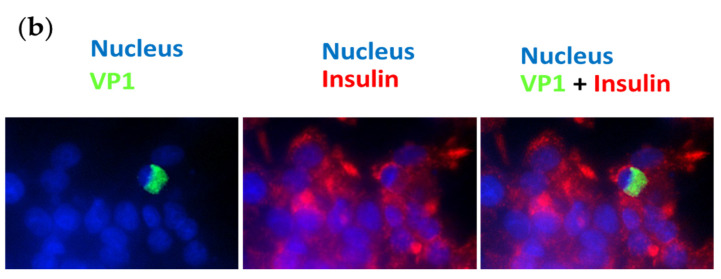
Pancreatic β cells persistently infected with CV-B4 E2 secrete insulin. Cultures of pancreatic β cells (INS-1 cell line) infected with CV-B4 E2 or uninfected were maintained for 90 days. (**a**) The cells were cultured for 2 h in RPMI-1640 medium, followed by the removal of the supernatant and cells. The presence of insulin in the culture supernatants of infected or mock-infected cells was evaluated using ELISA. The amount of insulin present in the culture supernatants was normalized by the total amount of DNA extracted from the cells. Values are the mean ± SD of 10 independent experiments. (**b**) CV-B4 infected INS-1 cells were fixed, permeabilized, and double-stained for intracellular VP1 (in green) and insulin (in red) with mouse anti-enterovirus VP1 and guinea pig anti-insulin antibodies using an indirect immunofluorescence assay. The nuclei (in blue) are stained with Hoechst Dye 33342^®^ (original magnification ×40). Pictures from one representative experiment out of three are shown.

The INS-1 β cells secrete insulin. The insulin concentrations detected using ELISA and normalized by the total amount of DNA extracted from cells were similar in the supernatant of infected and mock-infected cultures (Figure 3a). Insulin was detected in CV-B4 E2-infected INS-1 cells using an immunofluorescence assay, and in particular, in cells positive for the detection of viral protein VP1 (Figure 3b).

**Figure 4:**
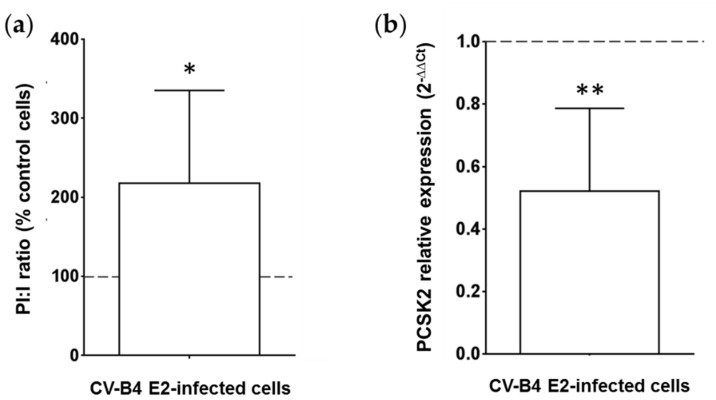
Profile of proinsulin, insulin, and pro-hormone convertase 2 in pancreatic β cells persistently infected with CV-B4 E2. Mock and pancreatic β cells (INS-1 cell line) persistently infected with CV-B4 E2 were cultured for 2 h in RPMI-1640 medium, and the supernatant was recovered. The cells were then lysed to extract total DNA and RNA (**a**) The concentrations of proinsulin and insulin in the supernatants of cultures were evaluated using ELISA. The ratios of proinsulin (PI) and insulin (I) concentrations were calculated. The results are expressed as % of the PI:I ratio compared with the PI:I ratio obtained with mock-infected cultures (control cells). The PI:I ratio of mock-infected cells is represented by a dotted line. Values are the mean ± SD of 10 independent experiments, * *p* < 0.05. (**b**) Pro-hormone convertase 2 (PCSK2) mRNA was quantified using RT-qPCR. Normalization was performed with β-actin mRNA. The relative expression in mock versus CV-B4 E2-infected INS-1 cells was determined with the 2^−ΔΔCt^ formula. The value of PCSK2 expression in uninfected cells is represented by a dotted line. The values are the mean ± SD of three independent experiments encompassing 4 to 6 replicates each, ** *p* < 0.001.

Insulin comes from the maturation of proinsulin. Intracellular proinsulin is cleaved into insulin, which is transported through vesicles to the extracellular medium. The proinsulin is nevertheless detectable in the supernatant in small quantity. A relationship between the concentrations of proinsulin and insulin in the culture can be used to assess the efficiency of conversion of proinsulin to insulin. The proinsulin and insulin concentrations in supernatants of infected and mock-infected cultures were measured using ELISA, and the ratio proinsulin/insulin (PI:I) was calculated. The PI:I ratio was more than 2.5 times higher in CV-B4 E2-infected INS-1 cells than in mock-infected cells (*p* < 0.05) (Figure 4a). PC2 is a pro-hormone convertase, encoded by the PCSK2 gene, responsible for the first cleavage of proinsulin during its maturation into insulin. The expression of PCSK2 mRNA was studied using RT-qPCR. PCSK2 mRNA was two times less expressed in INS-1 cells persistently infected with CV-B4 E2 than in control cells (*p* < 0.001) (Figure 4b).

### 3.3. Persistent CV-B4 E2 Infection of Pancreatic β Cells Changes the Expression of Cellular Proteins

CV-B4- and mock-infected INS-1 cells were lysed with buffer containing 1% SDS. The proteins of the cell lysate were analyzed using mass spectrometry, with the shotgun method. The presence of cellular proteins was evaluated by the SEQUEST score. Nearly 1700 different proteins were detected, including nearly 500 with a modified score in persistently infected cells. The identification score was considered to be modified when it was at least 1.5 times higher or lower than the score of the controls. The variation in the SEQUEST score of 47 proteins was more significant in cells persistently infected with CV-B4 E2 than in control cells (Table 2). A decrease in the score was observed for 45 proteins with degrees of statistical significance when compared with that of the controls, ranging from *p* < 0.0005 for 3 of them to *p* < 0.005 for seven others and *p* < 0.05 for the remaining 35. The score for seven proteins among 47 was zero in the infected cultures. By contrast, an increase in the score was observed for two proteins in the infected cultures: the FAM234A protein (*p* = 0.02) and isoform 2 of the HNRNP A3 protein (*p* = 0.02). Insulin and pro-hormone convertase 2 was not detectable with our method of mass spectrometry in the cultures of mock-infected cells and infected cells.

### 3.4. Persistent CV-B4 E2 Infection of Pancreatic β Cells Causes Changes in DNA Methylation

**Figure 5:**
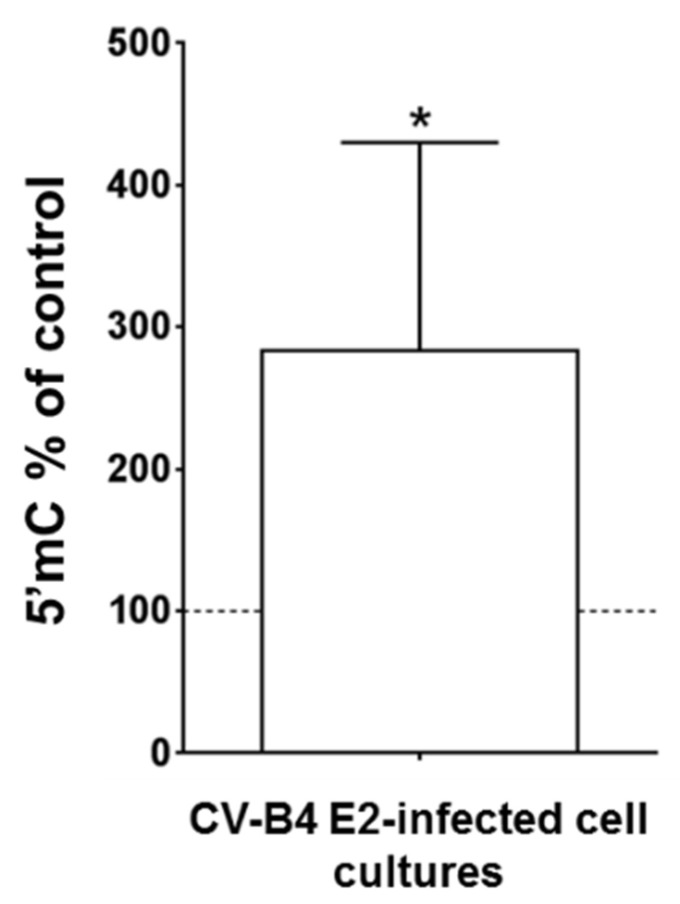
The DNA of pancreatic β cells persistently infected with CV-B4E2 was hypermethylated. Pancreatic β cells (INS-1 cell line) persistently infected with CV-B4 E2 or mock-infected were lysed to extract total DNA. The level of methylated cytosines (5′mC) in DNA of infected and mock-infected cells was measured using ELISA. The results are expressed as % of the 5′ mC level in DNA of mock-infected cells. The methylation value of mock-infected cells is represented by a dotted line. The values are the mean ± SD of four independent experiments, * *p* < 0.05.

DNA of mock and CV-B4-infected INS-1 cells was extracted and the level of methylated cytosines was measured using an ELISA-like assay. The level of methylated cytosines in DNA of persistently infected cells was significantly higher than that in DNA of control cells (*p* < 0.05) (Figure 5). In contrast, there was no significant difference in DNA methylation of CV-B4-infected INS-1 cells and mock-infected cells on day 2 p.i.

## 4. Discussion

We previously demonstrated the persistence of CV-B4 in human pancreatic islets [28] in human primary ductal pancreatic cells [38] and in a human β cell line (1.1 B4) produced by fusion of human pancreatic β cells with a human pancreatic duct cell line (Panc-1) [29]. This latter model of β cell does not produce insulin; therefore, it is not suitable for investigating the impact of virus persistence on the production of this hormone. In the present study, we report the persistent infection with CV-B4 of insulin-secreting β cells (INS-1 cell line).

The persistent infection with CV-B4 was established in β cell line INS-1 after an acute and lytic infection during the first few days p.i, followed by a stable release of infectious particles by surviving cells, which are morphologically comparable with mock-infected cells. The pattern of CV-B4 infection in our β cell system, comprising a lytic phase followed by virus persistence with release of infectious virions without cell lysis, is comparable to the one observed previously in the course of the infection of pancreatic ductal cells (Panc-1 cell line) and β cells (1.1B4 cell line) with CV-B4 [29,33]. The levels of infectious particles in supernatants of infected cultures collected during the weeks and months of follow-up varied from 10^3.5^ to 10^6^ TCID_50_.mL^−1^. Throughout the monitoring of the cultures, the percentage of cells harboring the VP1 capsid protein, detected using immunofluorescence assay, was low (≈1%). We previously showed using a single-cell RT-PCR that viral RNA was present in more than 50% cells of the human pancreatic duct cell line and human β cell line persistently infected with CVB4 E2, while the VP1 protein was only present in less than 5% cells [29,33]. Therefore, in our experiments, the hypothesis that the proportion of β cells persistently infected with CV-B4 harboring viral RNA is greater than that indicated by the presence of the VP1 protein cannot be excluded.

Pancreatic β cell infection with echovirus 16 and 30 and the impact on insulin secretion have been reported. In this model of acute infection, a decrease in insulin secretion was demonstrated [39]. Recently, it was shown that insulin mRNA expression and C-peptide levels were inhibited by persistent CV-B4 E2 infection in human primary pancreatic ductal cells differentiated into ICA in vitro [38]. Furthermore, Yin et al. reported that persistent infection of human pancreatic islets with the strain CV-B4 VD2921 did not affect the proinsulin and insulin content of β cells [40]. In agreement with this study, the amount of insulin secreted by β cell line INS-1 was not affected by persistent CV-B4 E2 infection in our system. However, insulin is impacted as the ratio of secreted proinsulin to secreted insulin is higher during infection, owing to a greater secretion of proinsulin than that in mock-infected cells.

We have shown that PCSK2 mRNA is downregulated in β cell line INS-1 persistently infected with CV-B4 E2. PCSK2 is the gene encoding PC2, which cleaves proinsulin between Lys 64 and Arg 65 residues. This cleavage separates the A and C chains of proinsulin. Maturation continues during vesicular transport and results in the secretion of insulin and C-peptide [2,3]. Inhibition of PCSK2 can disrupt insulin processing, resulting in increased secretion of proinsulin from β cells [4]. Exposure of β cells to IFN-α, which induces ER stress, has been reported to inhibit PC2 expression and thus disrupt insulin metabolism, resulting in excessive proinsulin secretion [41]. Owing to chromosomal rearrangements, it is believed that continuous cell lines do not produce IFN-α [42] even though IFN-α mRNA and protein can be detected in some of them [43,44]. It is not excluded that IFN-α or other cellular mediators released by infected cells in our model play a role in the alteration of insulin secretion we observed. Nevertheless, the hypothesis of a direct impact of the virus replication in the cell and/or of the presence of viral proteins and/or RNA on the metabolism of insulin should be explored. Interestingly, an increase in serum proinsulin has been reported in patients with T1D, and that precedes the disturbance of glucose homeostasis [4,45]. Furthermore, proinsulin is an autoantigen recognized by CD4^+^ T lymphocytes in patients with T1D [46,47,48]. Excessive proinsulin secretion is due to exposure of β cells to IFN-α or defective insulin maturation [41]. Further studies are needed to determine whether the alteration in insulin metabolism observed in patients is related to a direct effect of CV-B4 or other enterovirus on β cells and/or an indirect effect via IFN-α produced in response to infection.

The downregulation of PCSK2 mRNA in INS-1 cells persistently infected with CV-B4 versus controls indicates that somehow this virus replicating in the cytoplasm can have an effect on the nucleus, evidenced by the inhibition of the PCSK2 gene. This observation is reminiscent of the one already reported by our team concerning the inhibition of PDX-1 expression when pancreatic cells are persistently infected with CV-B4 E2 [33]. Furthermore, the inhibition of genes related to inflammation or to cellular physiology and metabolism in models of acute infection with CV-B3 and EV-71 has been reported in vitro as well [49,50]. We observed an overall DNA hypermethylation in β cell line, INS-1, cultures persistently infected with CVB4. This observation suggests that CV-B4 infection has an impact on the nucleus of cells and, in particular, on DNA. DNA methylation is associated with the regulation of gene expression. It is not excluded that the effect of the infection on DNA that we observed plays a role in the alteration of the expression of PCSK2 and other genes. Further studies are required to explore this hypothesis. The effect on the nucleus, and in particular on DNA, is intriguing as CV-B4 is an RNA virus whose viral cycle takes place in the cytoplasm. It is not excluded that this is an indirect effect, due to cellular mediators, but the hypothesis of a direct effect of the virus cannot be ruled out because it has already been reported that proteins of viruses belonging to the *Picornaviridae* family can localize to the nucleus [51]. Indeed, the VP1 protein of CV-B3 has a functional nuclear localization signal [52], and in cells infected with the foot and mouth disease virus belonging to the genus *Cardiovirus*, the viral protease 3C is able to cleave histone H3 in the nucleus [53,54].

Persistent infection of β cell line INS-1 with CV-B4 causes changes in insulin metabolism. In addition, the persistent infection has an impact on many β cell proteins as shown using shotgun mass spectrometry in our study. This method has already been used by other teams to characterize the proteome in bronchoalveolar lavage of patients infected with HIV or the proteome of myocardium of patients with dilated cardiomyopathy [55,56]. Shotgun mass spectrometry with SEQUEST score analysis identified nearly 1,700 proteins in INS-1 cells during this study. The identification score of nearly 500 proteins was altered in infected cells versus controls. The difference in scores was significant for 47 of them. This is the case of EIF4G, the score of which reduced, which is consistent with the notion of cleavage of this protein by the viral protease 2A of enteroviruses, the consequence of which is the inhibition of the translation of cellular proteins [57]. This is also the case of isoform 2 of HNRNP A3, the score of which increased [58] and which can interact with cellular and viral RNAs to facilitate infection by poliovius [59]. Shotgun mass spectrometry demonstrated a reduction in the score of the Prph, Dbn1, and Map1A proteins in particular, in the cultures of cells infected with CV-B4 E2. The impact of the infection on these proteins may play a role in the insulin abnormalities that we have observed since they are involved in vesicular transport and cytoskeletal structure, and it has been suggested that the dysfunction in the transport of insulin granules can alter insulin maturation [60].

Shotgun mass spectrometry provides a great deal of information concerning the profile of cellular proteins in β cell line INS-1 infected with CV-B4. This approach opens up prospects for studying the cellular consequences of infection; however, other strategies retain their interest, since the disturbances in insulin metabolism (Insulin/proinsulin ratio and PCSK2 inhibition) in the present study were demonstrated by ELISA and real-time RT-PCR.

In our model of persistent CV-B4 E2 infection of β cell line, abnormalities in cellular functions, proteins, and DNA were observed. Insulin maturation defect and inhibition of pro-hormone convertase involved in post-translational modifications of proinsulin were demonstrated. The impact of CV-B4 infection on insulin maturation with the release of proinsulin by β cells opens a new avenue concerning the viral pathogenesis of diabetes, as proinsulin plays a role in the development of autoimmunity against β cells [46,47,48]. It remains to be determined whether the epigenetic impact of CV-B4 on β cells can alter insulin maturation. Future studies will be directed along this line in our laboratory.

## Figures and Tables

**Table 1:** Oligonucleotide primers.

Target		Sequence
β-actin	*Forward*	5′-GGCACTCTTCCAGCCTTCCT-3′
	*Reverse*	5′-GCAATGCCAGGGTACATGGT-3′
CV-B4 E2	*Forward*	5′-CAAGCACTTCTGTTTCCCCGG-3′
	*Reverse*	5′-ATTGTCACCATAAGCAGCCA-3′
PCSK2	*Forward*	5′-CGAAACCAGCTTCACGATGAG-3′
	*Reverse*	5′-ACGCCGGCTTAGCAAAATGGA-3′

**Table 2:** Mass spectrometric analysis of the proteins of INS-1 cells persistently infected with CV-B4.

Accession	Protein	Infected Cells Score	Controls Score	Score Difference	*p*-Value
P21807	Peripherin	0.00 ± 0.00	12.56 ± 4.10	−12.56	***
Q07266-2	Isoform E1 of Drebrin	6.29 ± 3.00	17.56 ± 2.90	−11.27	***
Q4QQS7	Protein Umps	0.49 ± 0.99	5.40 ± 1.14	−4.91	***
P34926	Microtubule-associated protein 1A	2.00 ± 1.37	14.30 ± 5.17	−12.3	**
Q5XIM9	T-complex protein 1 subunit beta	7.86 ± 3.07	15.90 ± 2.48	−8.04	**
P23514	Coatomer subunit beta	1.05 ± 2.10	5.01 ± 1.13	−3.96	**
D4A2G9	Protein Ranbp1	0.00 ± 0.00	3.60 ± 1.79	−3.6	**
G3V7N5	Carnitine O-palmitoyltransferase 2, mitochondrial	0.00 ± 0.00	3.38 ± 1.66	−3.38	**
D3ZMY7	Protein Nt5c2	0.43 ± 0.87	3.75 ± 1.12	−3.32	**
F1LMC7	Septin-7	0.44 ± 0.87	2.60 ± 1.37	−2.16	**
A0A0G2K1J5	Plectin	4.04 ± 2.38	21.11 ± 13.47	−17.07	*
Q6URK4	Heterogeneous nuclear ribonucleoprotein A3 (HNRPA3)	0.00 ± 0.00	16.28 ± 13.66	−16.28	*
P12785	Fatty acid synthase	6.78 ± 3.89	22.06 ± 8.44	−15.28	*
A0A0G2JU82	Microtubule-actin cross-linking factor 1	7.59 ± 5.23	22.14 ± 9.29	−14.55	*
Q05982	Nucleoside diphosphate kinase A	6.26 ± 8.23	20.65 ± 6.46	−14.39	*
Q6URK4-2	**Heterogeneous nuclear ribonucleoprotein A3 (HNRPA3) isoform 2**	18.03 ± 4.53	4.98 ± 8.08	**+13.05**	*
A0A0G2K013	Alpha-actinin-4	9.04 ± 1.59	21.08 ± 7.58	−12.04	*
P06687	Sodium/potassium-transporting ATPase subunit alpha-3	0.00 ± 0.00	11.90 ± 7.78	−11.9	*
D4AD15	Protein Eif4g1	4.66 ± 2.13	16.16 ± 6.89	−11.5	*
A0A0G2K0Q7	Protein Mylk	3.17 ± 2.13	11.74 ± 4.47	−8.57	*
O35314	Secretogranin-1	5.04 ± 1.57	13.22 ± 4.74	−8.18	*
Q62667	Major vault protein	6.15 ± 2.75	14.06 ± 4.71	−7.91	*
D4AC23	Protein Cct7	6.14 ± 1.77	13.98 ± 4.59	−7.84	*
P04692-5	Tropomyosin alpha-1 chain isoform 5	1.62 ± 3.23	8.57 ± 5.17	−6.95	*
Q3MIE4	Synaptic vesicle membrane protein VAT-1 homolog	2.34 ± 2.20	9.00 ± 3.48	−6.66	*
D3ZRM9	Uncharacterized protein	0.00 ± 0.00	6.54 ± 3.76	−6.54	*
D3ZRM9	Uncharacterized protein	0.00 ± 0.00	6.54 ± 3.76	−6.54	*
Q62950	Dihydropyrimidinase-related protein 1	2.41 ± 3.70	8.67 ± 4.13	−6.26	*
F1MAA1	Ubiquitin-specific peptidase 47	2.26 ± 2.61	8.50 ± 4.06	−6.24	*
O35303-6	Dynamin-1-like protein isoform 6	1.91 ± 3.83	7.53 ± 2.90	−5.62	*
P41562	Isocitrate dehydrogenase [NADP] cytoplasmic	2.57 ± 3.14	8.07 ± 2.98	−5.5	*
D4A0C3	Protein Hid1	1.38 ± 1.78	6.44 ± 2.39	−5.06	*
A0A0G2JZ60	Protein Fsd1l	3.86 ± 3.10	8.89 ± 2.01	−5.03	*
A0A0G2JUN7	Thioredoxin reductase 1, cytoplasmic	2.77 ± 1.97	7.38 ± 2.33	−4.61	*
D3ZVQ0	Protein LOC100911959	2.72 ± 2.66	7.14 ± 1.73	−4.42	*
O70593	Small glutamine-rich tetratricopeptide repeat-containing protein alpha	1.03 ± 1.21	5.43 ± 2.75	−4.4	*
P50475	Alanine--tRNA ligase, cytoplasmic	0.44 ± 0.88	4.76 ± 2.41	−4.32	*
P27008	Poly [ADP-ribose] polymerase 1	1.90 ± 2.77	6.15 ± 2.62	−4.25	*
B2RYI2	Signal recognition particle subunit SRP68	2.03 ± 1.60	6.04 ± 1.87	−4.01	*
Q05096-3	Unconventional myosin-Ib isoform 3	1.96 ± 2.70	5.89 ± 1.41	−3.93	*
O88321	Antisecretory factor	1.47 ± 1.86	5.33 ± 2.30	−3.86	*
B2GV74	Kinesin light chain 2	1.08 ± 2.16	4.89 ± 2.36	−3.81	*
Q9Z1W6-4	Protein LYRIC isoform 4	0.54 ± 1.08	3.58 ± 2.25	−3.04	*
A0A0H2UHW4	PEST proteolytic signal-containing nuclear protein	0.42 ± 0.85	3.29 ± 2.02	−2.87	*
E9PT23	Putative sodium-coupled neutral amino acid transporter 10	0.46 ± 0.93	3.00 ± 1.92	−2.54	*
D3Z8U5	Metalloendopeptidase	0.87 ± 1.73	3.10 ± 1.25	−2.23	*
Q5M7W6	**Protein FAM234A**	5.24 ± 0.98	1.88 ± 2.18	**+3.36**	*

Mock and CV-B4-infected INS-1 β cells were lysed in a buffer containing SDS to extract the proteins. The proteins were analyzed using Shotgun mass spectrometry. The results are expressed as the SEQUEST protein identification score. The difference in score between infected cells and controls was calculated. Proteins from infected cells for which the identification score value is 0 are underlined. Results are presented as the mean ± standard deviation of six replicates. *: *p* < 0.05, **: *p* < 0.005, ***: *p* < 0.0005. In bold: score difference > 0. Unerlined: infected cells score = 0.
